# Testing the applicability of Watson’s Green Revolution concept in first millennium ce Central Asia

**DOI:** 10.1007/s00334-023-00924-2

**Published:** 2023-05-12

**Authors:** Basira Mir-Makhamad, Robert N. Spengler

**Affiliations:** 1 Department of Archaeology, Max Planck Institute of Geoanthropology, Kahlaische Straße 10, 07745 Jena, Germany; 2 Ancient Oriental Studies Department, Friedrich Schiller University, Zwätzengasse 4, 07743 Jena, Germany; 3 Domestication and Anthropogenic Evolution Research Group, Max Planck Institute of Geoanthropology, Kahlaische Straße 10, 07745 Jena, Germany

**Keywords:** Crop diffusion, Islamic conquest, Arboriculture, Irrigation, Sogdians, Trade, Urbanization

## Abstract

Drawing on archaeobotanical evidence from the central regions of Central Asia, we explore crop diffusion during the first millennium ce. We present a comprehensive summary of archaeobotanical data retrieved from this region dating to this period in order to better understand cultural drivers pushing agricultural intensification and crop diversification. We use these data to evaluate the applicability of Watson’s concept of a Medieval Green Revolution. Despite ecological limits to cultivation of most of Watson’s crops in much of Central Asia, some of these crops, particularly *Gossypium arboretum/herbaceum* (cotton) and *Oryza sativa* (rice), are prominent in the region today. In both cases there is now good archaeobotanical evidence showing that they were cultivated prior to the Islamic conquests in Central Asia. Moreover, the occurrence of several crops – *Solanum melongena* (eggplant), *Ficus carica* (fig), *Morus alba/nigra* (mulberry), and two spices – *Rhus coriaria* (sumac) and *Coriandrum sativum* (coriander) – have first been observed in phases dated to the 19th century ce. There is reason to believe that elaborate irrigation systems and seasonal rotation cycles were already in place in this region prior to the development of a centralized Arabic and Islamic government and are likely tied to urbanization in the first half of the first millennium ce. We suggest that most of the trappings of Watson’s model were present prior to this, but, as Watson’s thesis is multifaceted their presence alone does not nullify the model. Additionally, the ecological and cultural diversity of Central Asia means that a proper evaluation of Watson’s model requires a regionally specific examination.

**Supplementary Information:**

The online version contains supplementary material available at 10.1007/s00334-023-00924-2.

## Introduction

Over the past two decades, with growing investment in archaeobotanical, zooarchaeological, and isotopic studies, much of the prehistory and history of agriculture in Central Asia has been clarified, starting with Neolithic expansions into the Kopet Dag, Turkmenistan, around the end of the seventh millennium bce (Harris 2010). From there, most Central Asian experts believe that cereal crops dispersed north and east via an Inner Asian Mountain Corridor (Frachetti 2012; Spengler et al. 2014). Recent discoveries suggest that this Neolithic expansion may have reached Tongtian Cave in what is now Xinjiang, China, by 3500 bce (Zhou et al. 2020), possibly with an earlier expansion of pastoralists into the mountains (Taylor et al. 2021). Archaeological data (Spengler et al. 2017; Chang 2018) suggest that economic shifts towards greater focus on sedentary agriculture took place in certain areas of Central Asia, such as the mountain foothill zones, by the late first millennium bce in Central Asia. Over the last decade, there has been an explosion of interest in the timing and routes of crop spread across Eurasia. These studies largely showcase Central Asia as the crossroads of the ancient world, facilitating dispersal of East Asian crops westward and southwest Asian crops eastward. Nearly all of this research has focused on archaeological contexts that date to more than two millennia ago (Miller 1993, 1999; Spengler and Willcox 2013; Motuzaite Matuzeviciute et al. 2015, 2018, 2020a, b; Spengler 2015; Miller et al. 2016; Spengler et al. 2016, 2018a; Yang et al. 2020; Zhou et al. 2020); leaving the first millennium ce largely unstudied. This is particularly problematic for a holistic understanding of Central Asian history, as the peak periods of Silk Road trade, urban development, and imperial expansion all occurred during the first and early second millennia ce. The data that we pool in this paper also show that prominent dispersals of perennial crops and the diversification of agricultural systems occurred during this broad period. In addition, this paper makes a first attempt at bringing the archaeobotanical data into discussions of Watson’s thesis and first millennium ce crop diffusion of the Old World.

Based on historical sources from across southwest Asia and the eastern Mediterranean, Watson (1974) concluded that the organized central authority of the late first millennium ce, following the Islamic conquests or political conversions to Islam, resulted in an agricultural revolution. As he was writing shortly after the peak of the (20th century) Green Revolution, he was clearly drawing on Boserupian or Wittfogelian ideas of demography, innovation, and political systems as being inseparably tied to modes of production (ideas that have been prominent in western scholarship since Smith and Marx, and in archaeology since Childe). He argued that a centrally controlled labour force and government – regulated and protected commerce and trade routes, resulted in an inflow of new crop types and varieties as well as more intensive agrarian technologies. He envisioned agricultural innovations spreading from the core areas of the caliphate, across North Africa and into the lands around the southern and eastern shores of the Mediterranean Sea. Watson’s concept of a Medieval Green Revolution was defined by the rise and spread of specific crops, mostly of semi-tropical South Asian origins, into the temperate and arid Islamic world. He recognized that some of these crops were likely already under cultivation in these regions prior to the Islamic conquests, but in those cases, he emphasized that their intensity of cultivation would have been increased after the supposed Green Revolution. His main argument was that the intensification of farming through collective labour projects and the expansion of arable land facilitated greater diversity in the crops grown, fostering seasonal crop rotations, demographic growth, greater urbanization, and movement of merchants, soldiers, travellers, and scholars (especially the Arab geographers) over long distances (Watson 1983). However, criticism has risen around Watson’s model, in some cases challenging outright his statements (e.g. Johns 1984; Decker 2009; van der Veen 2011; Squatriti 2014; Amar and Lev 2017; Fuks et al. 2020).

**Fig. 1:**
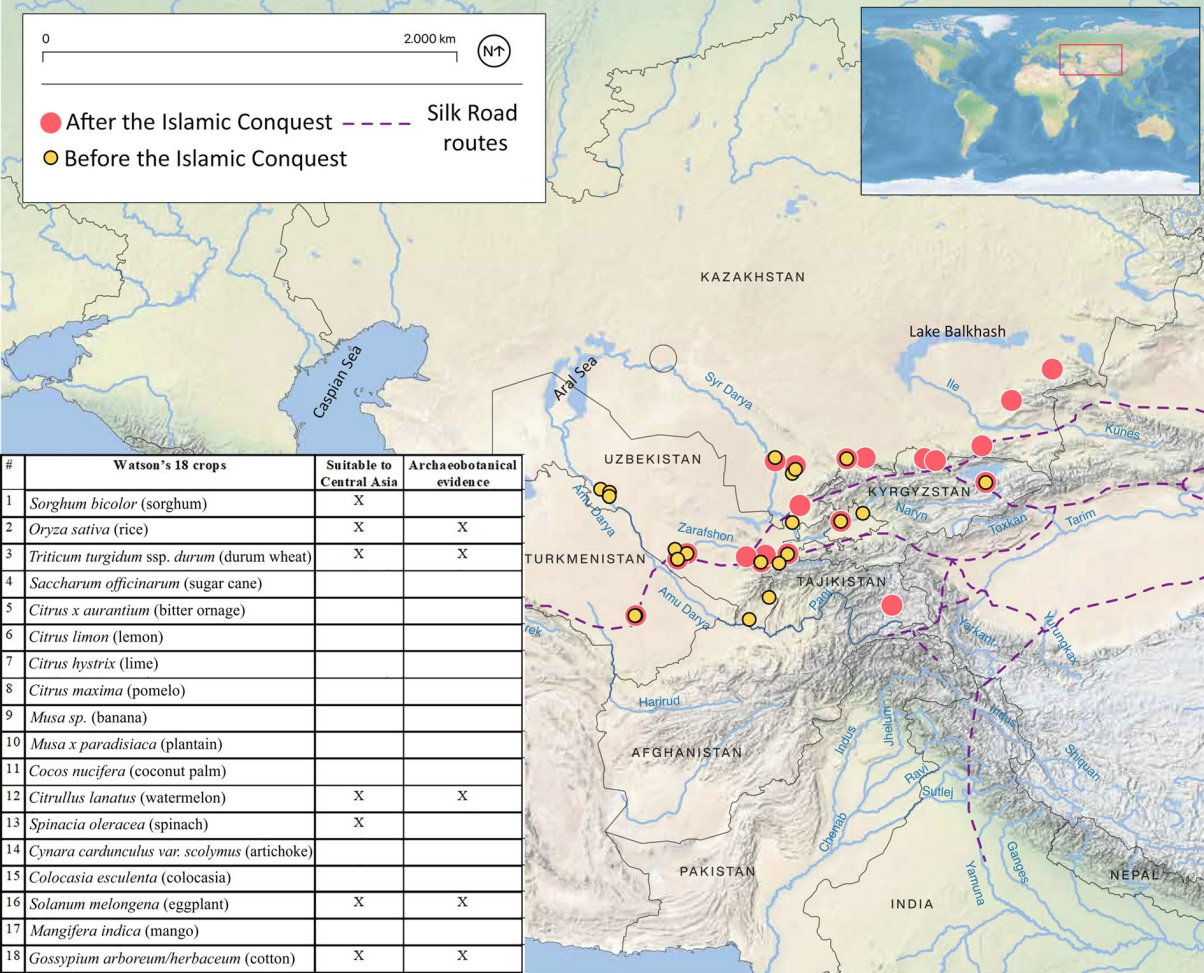
Sites with first millennium ce archaeobotanical data from Central Asia; inset-table with Watson’s 18 crops, marking those that are suitable for growing in Central Asia and those recovered in the region

In this study we are confronted with two more nuanced questions: (1) can we tie any of these crop introductions to the Islamic conquest of Central Asia; and (2) are associated cultural changes (demographic, political, artistic, and scientific) the result of agricultural intensification or a prerequisite for it? While we do not expect to answer these multifaceted questions in this modest study, we do hope to lay the foundations for future research and hopefully encourage Central Asian archaeologists to engage in Big-History debates. Indeed, current archaeological evidence suggests that the first millennium ce witnessed economic change, likely tied to urbanization, the introduction of new religions, greater labour investment in irrigation, more expansive commerce and exchange networks, and centralized political authority. Our objectives are to explore the history of new plant introductions and diversification in Central Asia in the first millennium ce. We seek to trace changes in agriculture from cultural contexts before and after the Abbasid Caliphate expansion into central and southern Central Asia (completed in 751 ce). We ask whether the introduction of Islamic political authority and Arabic legislature was a major driving force underlying agricultural development and crop diversification. We present archaeobotanical data from the central regions of Central Asia recovered from 36 sites (Fig. 1); however, since some sites reveal multiple phases of occupation, we consider the total number of datasets for this study to be 47 (ESM 1 and 2). In addition to archaeobotanical studies, we review old archaeological and art historical reports from the region, which mention plant remains or artistic representations of plants.

### An agricultural revolution in Central Asia?

While Central Asia was not directly part of Watson’s model, he did note repeatedly that the innovations of the core regions of the Islamic empires would have spread into neighbouring regions. While considering the applicability of Watson’s ideas in Central Asia, we first recognize that climate and local environmental factors (ESM 3), would have been obstacles for the cultivation of South Asian crops. Watson’s 18 plants (table in Fig. 1) served as measurable proxies for agricultural intensification, because identifying intensification in the archaeological record remains challenging. Islamic Central Asia according to Watson included Kashgar, Turfan, Khotan, and Lop-Nor, but some of his statements about irrigation and diffusion of plants were specific to the areas Transoxiana and Turkestan. Additionally, the conquest of the Caliphate into Central Asia appears to have taken different political forms in different regions, with Persian and Arab peoples promoting Islamic authority, but tolerating multi-ethnic and religious diversity; hence we use the term Islamic conquests here loosely to describe the gradual conversion to Islam and political shift to Arabic. Central Asia is and was a culturally and environmentally diverse region, and any model of economic development is unlikely to be applicable for it all, so we focus on the core areas from Fergana to the Khorezm Oasis.

## Crop diversification and Watson’s 18

Watson (1974, 1983) claimed that the new administrative systems were successful in acclimatizing several subtropical crops to northern conditions. That said, simple biological constraints would have made most of Watson’s 18 impossible to grow in the regions we are discussing; only *Gossypium arboretum/herbaceum* (cotton), *Oryza sativa* (rice), *Sorghum bicolor* (sorghum), *Triticum turgidum* ssp. *durum* (free-threshing tetraploid durum wheat), *Citrullus lanatus* (watermelon), *Solanum melongena* (eggplant), and *Spinacia oleracea* (spinach) would have been feasible to grow at this time in this region. Based on the plant remains recovered through flotation and dry-sieving (archaeobotanical studies) and randomly handpicked finds of plant remains during archaeological expeditions, we elaborate on the evidence for cultivation of these five crops in Central Asian oases. Spinach and sorghum remain unattested, in the case of the former likely due to preservation issues. Ultimately, testing the model on Central Asia does not affect its applicability in the core regions of the Caliphate, where Watson intended the model to be used.

### *Triticum turgidum* ssp. *durum* (durum wheat)

During the Neolithic in southern Central Asia, diploid and tetraploid glume wheats were cultivated (Harris 2010; Miller 2011), but by the fifth millennium bce, the glume wheats were almost completely replaced by free-threshing forms of hexaploid bread wheat, *T. aestivum* (Miller 2011). Tetraploid wheat rachises were reported at the Argyrzhal-2 site in Kyrgyzstan dating to the first half of the second millennium bce (Motuzaite Matuzeviciute et al. 2017). *Triticum aestivum* remained prominent across Central Asia and appear to be the only form of wheat to spread into East Asia in antiquity. However, at some point in the more recent past, *T. turgidum* ssp. *durum* spread into Central Asia and became a regular part of the cultivation system, with specific regional forms evolving, such as Khorezam wheat. Archaeobotanically, *T. turgidum* ssp. *durum* remains largely invisible in Central Asia, and it is unclear if this is a result of scholars misidentifying it as *T. aestivum* or due to a lack of investigation into more recent time periods. Many urban sites lack wheat rachises, presumably due to off-site processing of cereals, and the rachises are essential for confident discrimination between the two species. So far, only two likely tetraploid wheat rachises were reported from Tashbulak (Spengler et al. 2018b), two possible rachises from Paykend (Mir-Makhamad et al. 2021), and one from Kok-Tosh (unpublished), all from occupation layers dated to the Qarakhanid period. In all three cases, variations or distortions in *T. aestivum* rachises cannot be ruled out. Due to the lack of comprehensive archaeobotanical studies in the region, we do not have enough evidence to discuss whether *T. turgidum* ssp. *durum* was present, introduced, or re-introduced at the time of the Islamic conquest in Central Asia.

### *Oryza sativa* (rice)

**Fig. 2:**
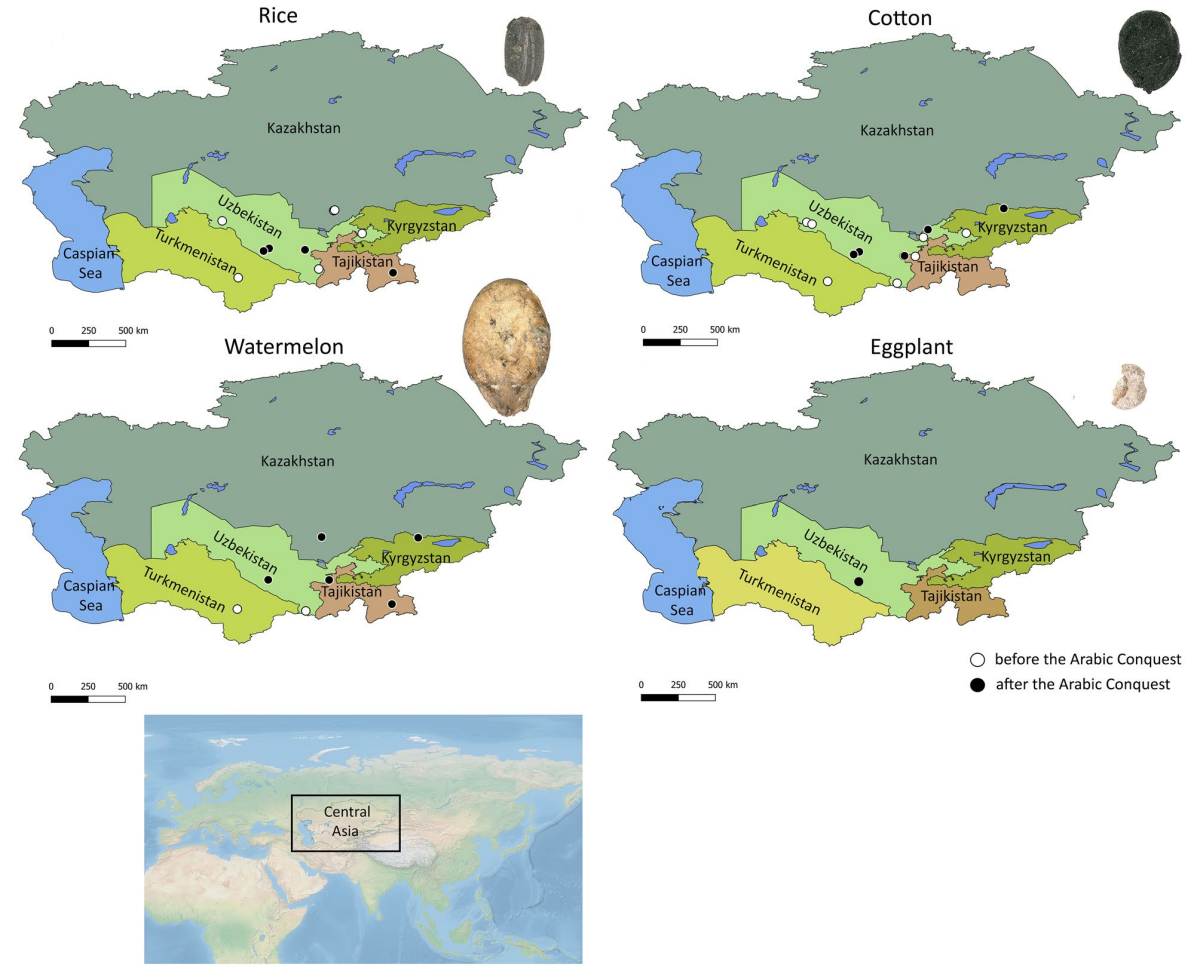
*Oryza sativa*, *Gossypium arboretum/herbaceum*, *Citrullus lanatus* and *Solanum melongena* remains from archaeological contexts in Central Asia before and after the Islamic conquest

Rice is a prominent annual crop in Central Asia today, being cultivated in Uzbekistan, southeastern Kazakhstan (Spengler et al. 2021b), the southwestern corner of Kyrgyzstan (Uzgen district), and northwestern Tajikistan (Panjakent district). Currently, the earliest archaeobotanical evidence for rice appears only at the terminus of the first millennium bce (Spengler et al. 2021b). Grains of rice have been recovered from several sites, including Erk-Kala (Usmanova 1963) and Teshik-Kala (Tolstov 1948) in Turkmenistan, Khalchayan (Chen et al. 2020) and Munchaktepa (Gorbunova 1986) in Uzbekistan, and Karaspan-Tobe and Djuvan-Tobe in Kazakhstan (Bashtannik 2007) – all of which date to before the Islamic conquests (Fig. 2a). Regarding pre-Islamic rice cultivation, Watson (1983, p 15) stated, “its cultivation in these regions, however, was probably limited”. While rice grains are rare from Central Asian archaeological sites, they do appear to increase in prominence during the late 9th–10th centuries. From the later medieval urban site of Bukhara, more than 200 mineralized grains were recovered from cesspits (unpublished), and 128 grains were recovered from the high-elevation mining settlement and eastern caravan stop of Bazar-Dara, in Tajikistan (Bubnova 1987). The counter variables here are that the Bukhara contexts represent an elite residence at the capital of the Samanid Empire, and both sites express exceptional preservation (mineralization and permafrost). Rice appears to remain a minor crop in other mercantile centres, such as Paykend (Mir-Makhamad et al. 2021) and Afrasiab (unpublished). Ongoing research around Panjakent (Tajikistan), where rice is intensively cultivated today, has, as of yet, not produced any ancient rice grains. Therefore, we speculate that rice cultivation was restricted to water-abundant oases and elite contexts in early Islamic Central Asia. In addition, some scholars have suggested the possibility that rice was not cultivated on a large scale until the past few centuries (Spengler et al. 2021b).

### *Gossypium arboreum/herbaceum* (cotton)

Today, cotton is grown across much of southern Central Asia; however, Uzbekistan has remained the main cotton producer for decades, followed by Tajikistan (FAOSTAT 2022). The occurrence of cotton seeds and textile fragments in the archaeological record demonstrates the development of cotton cultivation in southern Central Asia by the first half of the first millennium ce. Brite et al. (2017) suggest that cotton production could have taken place already by the 3rd–5th centuries ce at the Kara-tepe site in northwestern Uzbekistan. In addition, our project in Bukhara proves that cotton was present in the oasis by the first half of the first millennium ce, with direct dating of cotton seeds ranging from 261 to 532 ce (a mean value of cal. 401 ce) (unpublished). Furthermore, cotton seeds have been found in many other pre-conquest sites, including Kainar (unpublished data), Panjakent (Mir-Makhamad et al. 2022b), and Mugh (Vasil’ev 1934; Danilevsky et al. 1940) in Tajikistan; Kyuyk-Tepe (Gorbunova 1986), Kanka (Buryakov 1990), Balalyk-Tepe (Al’baum 1960), and Teshik-Kala (Tolstov 1948) in Uzbekistan; and Merv in Turkmenistan (Herrmann et al. 1993; Herrmann and Kurbansakhatov 1994) (Fig. 2b).

Archaeologists working at Panjakent argued that cotton and possibly silk textile production was already practiced in the 7th and beginning of the 8th centuries ce (Belenitsky et al. 1973). Furthermore, mural paintings from Balalyk-Tepe, Varakshi, Panjakent, and Afrasiab demonstrate that fabric patterns varied between cities and regions (Belenitsky et al. 1973), potentially demonstrating different local textile manufacturing traditions across Sogdiana. More than 100 fragments of cotton textiles belonging to at least 16 clusters were recovered from the Mugh fortifications in the Upper Zeravshan Basin of Tajikistan (Yakubov 1979), which were abandoned during the Islamic conquests in 722 ce. The specialists studying the cotton textiles recovered from Mugh concluded that they were locally produced for sackcloth, clothes, and stockings. In addition to fabric fragments, archaeologists recovered parts of a loom (Vinokurova 1957). Cotton fabrics were also found in the cemeteries of the Isfara group in Fergana (Gorbunova 1986). Historic sources (e.g. Narshakhi 943) also attest to Bukhara becoming a centre of cotton textile production in the 9th century ce. There is solid archaeobotanical evidence showing that cotton was present along the Syr-Darya River and across central and southern Central Asia prior to the Islamic period. Although, again, Watson (1983) qualified his statement, noting that people were likely familiar with cotton in the first half of the first millennium ce from Mongolia to Turkistan.

### *Citrullus lanatus* (watermelon)

*Citrullus lanatus* is extensively cultivated across Central Asia today, often as a complement to *Cucumis melo* (sweet melon). While there are medieval artistic depictions of *C. melo* and *Punica granatum* (pomegranates) from Central Asia (e.g. Al’baum 1975; Shishkina 1979; Azarpay 1981; Hensellek 2019), there are no clear representations of ancient watermelons, leaving open the question of when they arrived in this part of the world. Ancient watermelon seeds have been reported from a few sites in southern Central Asia dating to before the Islamic conquest, including Erk-Kala in Turkmenistan (Usmanova 1963) and Balalyk-Tepe in Uzbekistan (Al’baum 1960), as well as shortly after the Islamic conquest, including at Kuiruk-Tobe in Kazakhstan (Bashtannik 2007), Bukhara in Uzbekistan (unpublished), Ak-Beshim in Kyrgyzstan (Nakayama and Akashi 2020), and Bazar-Dara (Bubnova 1987) and Tirmizak-Tepa (Negmatov et al. 1973) in Tajikistan (Fig. 2c). None of these seeds have been radiocarbon dated, and the only ones with a secure context and confirmed identifications come from Bukhara, dating to the Samanid period (unpublished). *Citrullus lanatus* seeds are rare in the archaeobotanical record and are primarily recovered from handpicked contexts in early archaeological reports – all of which require further verifications. Archaeobotanical remains of mineralized seeds have been recovered from three sites: Bukhara (cesspits), Kuiruk-Tobe (cesspit) (Bashtannik 2007), and in Ak-Beshim (Pit 3) (Nakayama and Akashi 2020), all dated to the 9th–11th centuries ce. *Citrullus lanatus* was described by a 13th century ce Chinese traveller (1228 ce, Bretschneider 1888) as a fragrant, sweet vegetable of enormous size growing in Samarkand.

### *Solanum melongena* (eggplant)

Li Chichang, a companion of Kiu Changchun, composed a narrative of their trip to Central Asia and southwest Asia by order of Genghis Khan in 1220, describing Samarkand as a land where purplish coloured eggplants shaped like a finger were grown (Bretschneider 1888). However, the only archaeobotanical evidence of *S. melongena* consumption in Central Asia comes from Bukhara, dated to the 10th–11th centuries ce. *Solanum melongena* remains were recovered in the cesspit of Shakhristan, which is located only 100 m from the Bukhara Arc. As *S. melongena* seeds are usually only recovered mineralized, it is likely that the preservation mode is biasing against the recovery of earlier specimens. For example tomato and pepper seeds are likely to survive human digestion (Lee et al. 2005); consequently there is a higher chance of recovering them from cesspits or latrines.

## Introductions in the first millennium ce

Amar and Lev (2017) state that several plants were overlooked or not given enough coverage by Watson. In Central Asia, there are a few possible introductions that might support Watson’s model, but they are all based on limited data. Due to the limited number of archaeobotanical studies from the period directly before the Islamic conquests, compounded by taphonomic issues, we avoid making any conclusions regarding other newly introduced plants into early Medieval Central Asia. Nevertheless, bearing in mind these limitations, we review some of the available findings to which archaeobotanists should pay additional attention in the future. Currently, data from the first half of the first millennium (1–400 ce) consist of 7 sites; while there are 19 sites with data from 401 to 750 ce, and 21 from 751 to 1275 ce.

### Fruits and nuts

**Fig. 3:**
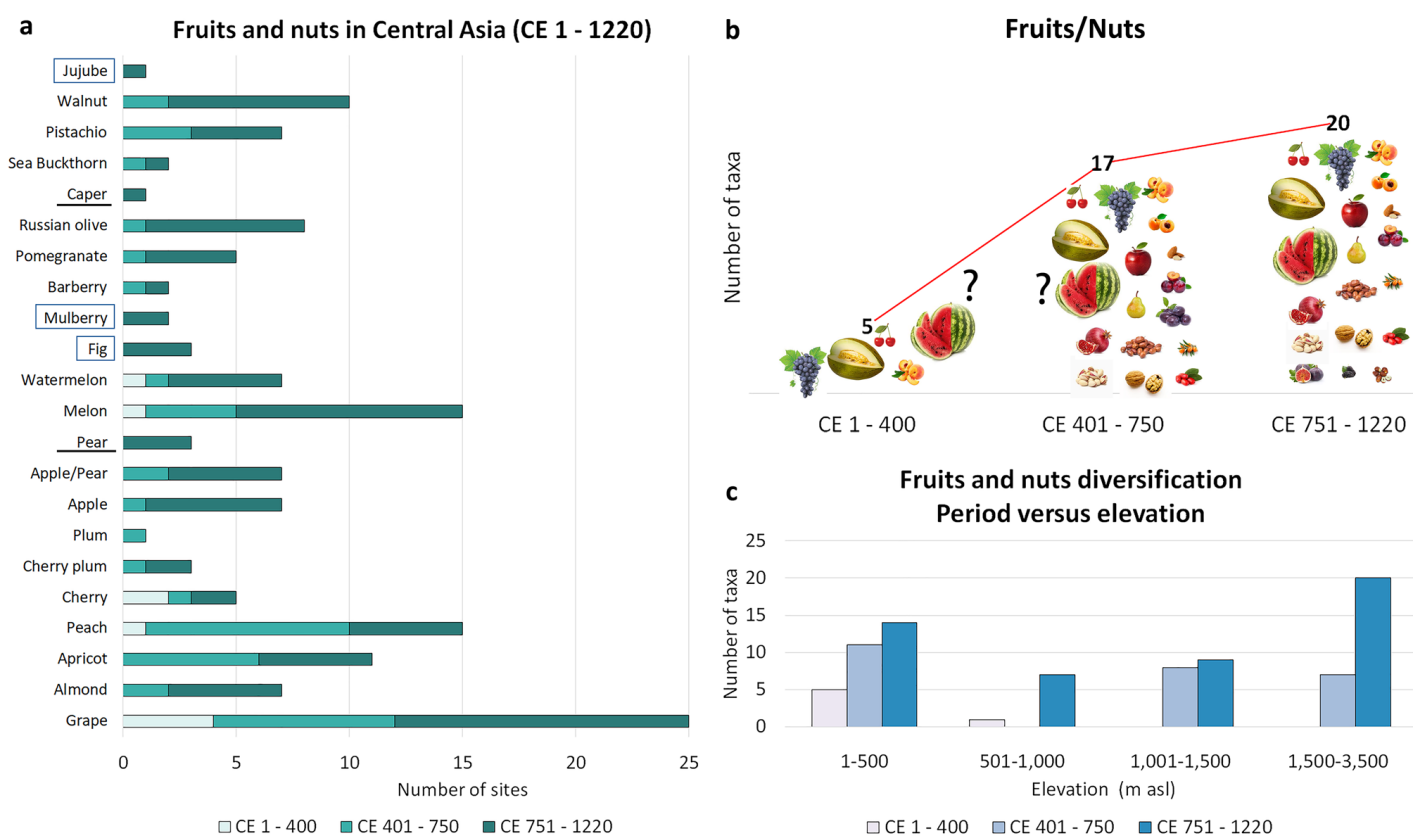
**a** fruit/nut intensification in Central Asia through time, **b** diachronic fruit/nut diversification, **c** fruit/nut diversification based on site elevation (ESM 2, list of sites and GPS coordinates)

By the end of first millennium bce, *Vitis vinifera* (grape) is the only woody perennial well attested in archaeological contexts. In contexts dating from 1 to 400 ce, seeds of *Vitis* (Usmanova 1963; Chen et al. 2020; Stark et al. 2022), melons, likely *C. lanatus*, *Prunus avium/cerasus* (sweet/sour cherry), and *Prunus persica* (peach) have also been reported (Usmanova 1963). We assume that all other fruits and nuts under cultivation were introduced or locally brought under cultivation in the central part of Central Asia after the 5th century ce and maybe a century later in the Middle Syr-Darya and northern Tian-Shan. The intensification of tree cultivation in the region appears to parallel urban development, a trend that has been suggested for Asia more widely (Dal Martello et al. 2023; Childe 1964; Fuller and Stevens 2019). There was a gradual increase in the ubiquity of nut shells, likely *Juglans regia* (walnut), *Pistacia vera* (pistachio) (Mir-Makhamad et al. 2022a), and possibly *Prunus dulcis* (almond), in early Islamic Central Asia (unpublished). In addition to nuts, fruits, notably *Cucumis melo*, *Citrullus lanatus*, *Elaeagnus angustifolia* (Russian olives), *Punica granatum*, *Malus domestica* (apple), *Pyrus* sp. (pear), and *V. vinifera*, appear to become more prominent prior to the 9th century ce (Fig. 3a). Hawqal Ibn (977, pp 237–238) described Sogdiana, Bukhara, and Fergana in the 10th century ce as regions with cultivated plains, full of gardens possessing all kinds of fruits and herbs.

In the early Islamic period, there seems to have been an increase in the number of fruit/nut species consumed; however, we face preservation biases since many types of fruit and nut remains only come from cesspits, e.g. Kuiruk-Tobe (Bashtannik 2007), Bukhara (unpublished), Kok-Tosh (ongoing project), and Novopokrovka (ongoing project). These contexts are rich in plant remains (mainly mineralized) and may bias discussions, as certain plants may simply not preserve in non-cesspit contexts (like *S. melongena* or spices). Mineralized *Ficus carica* (fig) achenes and *Morus* sp. (mulberry) seeds were recovered via sediment flotation from cesspits at Bukhara and Kok-Tosh. The same situation has been observed in other regions, such as Jerusalem (Amichay et al. 2019), where scholars (Fuks et al. 2020) also question if these finds represent a signal of innovation or preservation. Bubnova (1987) also reported *Ficus* at the high-elevation mining and caravanserai settlement of Bazar-Dara, located at 3,943 m a.s.l., a combination of desiccation and permafrost allowing for its preservation. *Ziziphus* sp. (jujube) stones in Central Asia were also only reported from Bazar-Dara (Bubnova 1987); earlier *Ziziphus* remains have been recovered to the east in Xinjiang from the second half of the first millennium bce (Jiang et al. 2013) and a small-seeded (likely a wild) local variety was recovered to the south in Swat, dating to the early first millennium bce (Spengler et al. 2021c). We also recognize the possibility for the exchange and short-distance movement of some of these plants, especially of dried fruits and nuts. The many fruit and nut remains recovered at Tashbulak and Bazar-Dara were found outside the parameters of their ecological constraints and clearly represent the transport of food to high elevations from the valleys below the villages.

### Legumes

Seven different pulses, including *Pisum sativum* (pea), *Lens culinaris* (lentil), *Cicer arietinum* (chickpea), *Lathyrus sativus* (grass pea), *Vicia faba* (fava bean), *Vicia sativa* (common vetch), and *Vigna* cf. *radiata* (mung bean) have been recovered from Central Asian sites dating to the first millennium ce. *Pisum*, *Lens*, and *Lathyrus* were introduced into Central Asia from the Iranian Plateau by the third millennium bce (Spengler 2019), although they remain rare in archaeological assemblages dating before the 5th century ce. There is more archaeobotanical evidence for legume consumption from the second half of the first millennium ce, when cultivation of *Lens* and *Pisum* became more prominent or culinary practices were changed. The earliest finds of *V. faba* (n = 2) date to the second millennium bce sites of Adji-Kui (Spengler et al. 2018a) and Togolok (Billings et al. 2022), located in the Murghab region of Turkmenistan. However these specimens are a microcarpic form, whereas a macrocarpic variety was clearly introduced in the medieval period. The large-seeded *V. faba* has been recovered from four archaeological sites in Tajikistan from prior to the Islamic conquests: Mugh (Danilevsky et al. 1940), Panjakent (Mir-Makhamad et al. 2022b), Kainar, and Sanjar-Shakh (ongoing study). In addition, we believe that Bashtannik et al. (2015) recovered *V. faba* instead of common beans as he claims (*Phaseolus* sp.; a North American domesticate) based on a reassessment of his published photos. *Vicia faba* remains were also recovered at Ak-Beshim in Kyrgyzstan (Nakayama and Akashi 2019) from contexts dating to the 10th century ce.

Currently the oldest *Vigna* cf. *radiata* recovered from Central Asia comes from the Balalyk-Tepe fortified settlement in Uzbekistan dating to the 4th-5th centuries ce (Al’baum 1960). Additionally, hundreds of *V.* cf. *radiata* beans were recovered from a khum, which likely functioned as an oven, in Bukhara. We directly dated one of the *V.* cf. *radiata* beans to between cal ce 663–775 (OS-165287), with a mean of cal ce 723 (unpublished). *Vigna* cf. *radiata* is also reported from the early Islamic site of Djuvan-Tobe in Kazakhstan (Bashtannik 2007) and from Bazar-Dara (Bubnova 1987).

### Spices

The first solid evidence for spices in Central Asia includes seeds from *Rhus coriaria* (sumac), a single possible *Piper nigrum* (black pepper), *Coriandrum sativum* (coriander), and *Sesamum indicum* (sesame), all of which come from cesspits at Bukhara, dating to the 9th-10th centuries ce (unpublished). In addition to Bukhara, *S. indicum* was recovered at Ak-Beshim and dated to the 10th-11th centuries ce (Nakayama and Akashi 2020). *Piper nigrum* originated in southwest India and is one of the most commonly used spices in Central Asia today. We avoid making any further conclusions regarding the one likely *Piper* seed; however, from the *Sogdian letters*, recovered in Dunhuang in 1907 by Aurel Stein, *Piper* was mentioned as one of the goods that Sogdians sold in the 4th century ce (Livšic 2009). *Rhus coriaria* could have been locally cultivated because it is widely distributed across southern Eurasia in the wild (Browicz 1982). However, the earliest *R. coriaria* remains come from southwest Asia (Haldane 1990; Fairbairn et al. 2002, 2019). *Coriandrum sativum* grows wild across much of West Asia. It was likely utilized for at least nine millennia according to data that were summarized by Zohary et al. (2012). In the same period of time *C. sativum* remains were recovered in the Tutankhamun’s tomb in Egypt where it does not grow wild (Zohary et al. 2012) and together with *R. coriaria* on the Uluburun ship dated to the 14th century bce (Pulak 2008).

## Urbanism and irrigation development

Watson envisioned irrigation systems falling into disrepair and silting up by the dawn of the Islamic Conquest, whereas the new political leaders repaired old irrigation works and constructed new ones. Indeed, historians have argued that more varieties of irrigation techniques to catch, channel, store, and lift water appeared in the Mediterranean and North Africa after the 7th century ce. Avni (2018, p 313) suggests that “the massive expansion of qanats was associated with the new geo-political situation following the Arab conquest”. Especially discussing Central Asia, Watson (1983, p 104) stated that limited areas in Transoxiana, “had elaborated irrigation works nearly all of which can be traced back to a remote antiquity”. Watson (1983, p 110) also envisions the *qanat* technique being invented in Iran and slowly diffusing to Central Asia and to North Africa only after the Islamic conquest, and likely southwestern Central Asia slightly earlier. However, recent studies dedicated to ground water catchment systems clearly illustrate that *qanat* technology was widely used by the first millennium bce in Egypt, southeast Arabia, and in Iran (del Cerro and Córdoba 2018; Charbonnier and Hopper 2018).

Based on data from Kafir-Kala, Afrasiab (Mantellini 2019), Paykend (Omel’chenko 2019), Vardanzeh (Mirzaachmedov et al. 2019), Panjakent (Marshak and Raspopova 2016), and Termez (Belenitsky et al. 1973), we suggest that significant urbanization in Sogdiana likely only began in the 4th century ce. Settlements such as Bukhara, Afrasiab, and Paykend, lack clear destruction levels associated with the Islamic conquest, and if the transition at these cities was violent, they were readily restored and appear, from an archaeological perspective, to have been continuously occupied until the Mongol conquests (Bukhara remains settled to the present). Mirzaachmedov et al. (2019) reported that Vardanzeh became a major urban settlement and a centre of agriculture after Arabic political authority took control. On the other hand, Mantellini (2017) demonstrates a rapid decrease of site numbers at the beginning of the 9th century ce around Samarkand, which can be interpreted as a centralization of urban power and inner population migration into urban centres, possibly tracing back to the Samanid period (ce 875–999), or an actual reduction of population. Archaeological data seem to suggest that a new wave of urbanization spread across Central Asia starting in the 9th and 10th centuries (Belenitsky et al. 1973). On the northern foothills of the Tian-Shan Mountains, intensive urbanization started in the 4th century ce, reaching its peak in the mid-9th to 12th centuries ce. The situation was different downstream of the Syr-Darya River, where the first settlements were already constructed by the end of first millennium bce, with a rapid increase in the mid-6th century ce and continuing to grow until the 12th century ce (Sala and Deom 2010). Based on these observations, urbanization was a continuous process, expanding in development roughly from southwestern to northeastern Central Asia through time, and presumably paralleling agricultural intensification.

Most scholars suggest that irrigation was already well developed before the Islamic conquests (Lewis 1966; Lisitsina 1969; Rapen 2010). Climatic variations (Clarke et al. 2005; Toonen et al. 2020) and political and economic dynamics (Brite 2016) served as regionally specific drivers of irrigation development and reduction. Some scholars (Dukhovny and de Schutter 2011) have linked the consequences of war in ancient Central Asia with a lack of water management, based on a perceived depopulation. Large-scale irrigation systems may have been established in many areas by the late first millennium bce, including Merv, Bactria, Sogdiana, Khoresm, Chardara, and Otrar to supply water to agricultural fields (Brite 2016). However, the construction of irrigation works took place at different times in different geographic zones and appears to have never been uniformly organized across Inner Asia, possibly until Russian Imperialism.

Several studies have been dedicated to the large-scale irrigation constructions around Afrasiab, where artificial channels (*sai*) were dug off of the Central Dargom canal (Shishkina 1994; Malatesta et al. 2012). Three artificial water reservoirs around Afrasiab were built between the 7th and 4th centuries bce (Vyatkin 1926; Akhmedov 2013); moreover, archaeologists report discovering many wells in the territory around the ancient capital (Vyatkin 1926). The Dargom diverted water from the south of the ancient city and the Bulungur from the north, both representing artificial banks of the Zeravshan River. There has been considerable debate over the dating of the Dragom (Shishkina 1994; Grenet 2002; de La Vaissière 2005; Mantellini et al. 2008; Malatesta et al. 2012; Mantellini 2015, 2018, 2019), with some scholars arguing for a genesis by the 5th century bce (Shishkina 1994) and others pushing for the early first millennium ce (Stride et al. 2009; Malatesta et al. 2012).

De la Vaissiere (2005) claims that the western periphery of the Bukhara Oasis was extended 22 km, due to irrigation development in the 6th century ce. Golden (2011) claims that since the Qarakhanid period, urban development and extensive irrigation systems were erected across the northern Tian-Shan, notably in Talas, Chui, and Otrar, a conclusion also drawn by other scholars (Bosworth and Asimov 2000). Clarke et al. (2005) described several periods of irrigation expansion in the Syr-Darya region: (1) the period between 200 and 650 ce in the Ortar Oasis and between 650 and 950 ce when people stored seasonal floodwater in natural depressions linked to the canals; (2) a period when “the first irrigation system developed on floodplains proximate to secondary branches of the Arys and Bogun delta and of the river Syr-Darya” (p 382); and (3) between 950 and 1200 ce, when irrigation was more organized but less extensive when the “water offtake structures were built on the main river course” (p 382). Toonen et al. (2020, p 32,987) studied the hydrological conditions of the Otrar Oasis (southern Kazakhstan) writing, “The Arab conquest took place at a time that was probably the most favorable for floodwater farming in the last millennia”, contrary to the situation Watson envisioned for other regions of West Asia.

## Conclusions

Given the limited available data, we remain cautious in our conclusions, but speculate that irrigation was gradually developing both before and after the Islamic conquests, and that the degree of labour investment in canal construction was not uniform across all regions of Central Asia. Archaeological investigations are not conclusive enough across Central Asia to verify whether this continuous economic growth took the form of a significant punctuated equilibrium or a continual gradualism. Additionally, there is some reason to believe that seasonal crop rotations were already practiced in some parts of Central Asia prior to the Medieval period. Cotton appears to have been well-established throughout southern pre-Islamic Central Asia, while rice seems to have been a minor crop before and remained so until more recently. Watermelons may have been introduced before the Islamic conquest, specifically in the southern zones; however, cultivation of the watermelons spread further to the north and east likely only after the conquest. The timing of the introduction of free-threshing tetraploid wheat and eggplant remains unclear. Looking beyond Watson’s 18, an increased prevalence of cultivated legumes and arboreal crops seems to parallel urbanization. The earliest archaeobotanical evidence for spices (coriander and sumac) dates to the 9th-10th centuries ce.

The hardest part of testing the Watsonian thesis is its lack of falsifiability; in the sense of Popper (1959), any scientific theory must be disprovable. Given that so many scholars in the social sciences are averse to parsimonious models of cultural development, the principle of falsifiability is often overlooked by archaeologists and historians. Spengler (2021) has noted elsewhere that the open-endedness of many theories in archaeology is the reason for their longevity, as well as the heated debates that they tend to provoke. Nonetheless, Watson’s hypothesis does not fit well within the core areas of Central Asia; this is due in part to the constraints to growth of the majority of Watson’s 18 in the north. More importantly, there simply were differences in the political systems, processes behind the expansion of the Islamic state, the degree of centralization of control, and development complex farming systems – both between Central and South Asia, as well as within Central Asia. Our discussion does nothing to discredit Watson’s core claims, as he intended them to primarily apply to the Mediterranean region; to the contrary, we hope to enrich discussions by building on the thesis.

The first millennium ce, as a whole, was likely a period marked by the first cultivation of cash-crops and increased exchange in Central Asia. For example, according to one historical source (Narshakhi 943), in the second half of the first millennium ce, there was a specialized area within the early-medieval bazaar of Bukhara solely for the sale of pistachio nuts. Ibn Fadlan, traveling from Baghdad to the Volga River via southwestern Central Asia in the 10th century ce, noted that dried fruits, nuts, millet and spices had been offered as gifts (Frye 2005). Hence, we believe that Watson was correct in his linking of intensification of exchange to urbanization and irrigation systems. However, the intensification and extensification of agricultural systems across Central Asia needs to be explored on a sub-regional scale, as the diverse mosaic landscape fostered an equally diverse repertoire of economic practices (Spengler et al. 2021a). Intensification and diversification in the northern Tian-Shan likely occurred concurrently with the Islamic invasion, but in the core areas – what was once Sogdiana – cultivation is widely attested from several centuries earlier than Watson proposed, but it may not have peaked until the 9th–10th centuries ce. Lastly, historical sources and archaeological data attest to Sogdian movement all over Central Asia during the 8th century ce, parallel to the Islamic conquest, further suggesting that a complex milieu of social factors were interacting (de la Vaissière 2017)**.**

## Supplementary Information

Below is the link to the electronic supplementary material.

Supplementary file1 (XLSX 21 KB)

Supplementary file2 (XLSX 13 KB)

Supplementary file3 (DOCX 30 KB)

## References

[CR1] Akhmedov M (2013) Paннecpeднeвeкoвый «дoм винa» нa Aфpacиaбe (Early Medieval “House of Wine” on Afrasiab). In: Lurie PB, Torgoev AI (eds) Sogdians, their precursors, contemporaries and heirs. The State Hermitage Publishers, St. Petersburg, pp 190–195

[CR2] Al’baum L (1960) Бaлaлык-Teпe (Balalyk-Tepe). Izd-vo Akadmiia Nayk Uzbekskoĭ SSR, Tashkent

[CR3] Al’baum L (1975) Живoпиcь Aфpacиaбa (Afrasiab murals). FAN, Tashkent

[CR4] Amar Z, Lev E (2017) Arabian drugs in medieval mediterranean medicine. Edinburgh University Press, Edinburgh

[CR5] Amichay O, Ben-Ami D, Tchekhanovets Y et al (2019) A bazaar assemblage: reconstructing consumption, production and trade from mineralised seeds in Abbasid Jerusalem. Antiquity 93:199–217. 10.15184/aqy.2018.180

[CR6] Avni G (2018) Early Islamic irrigated farmsteads and the spread of *qanats* in Eurasia. Water Hist 10:313–338. 10.1007/s12685-018-0225-6

[CR7] Azarpay G (1981) Sogdian painting: the pictorial epic in oriental art. University of California Press, Berkeley

[CR8] Bashtannik S, Voyakin D, Buranbaev R (2015) Apxeoбoтaничecкиe иccлeдoвaния цитaдeли cpeднeвeкoвoгo гopoдищa Tapaз в 2014 г. (Archaeobotanical Studies of the Citadel of the Medieval Settlement Taraz in 2014). Bull Archaeol Anthropol Ethnogr 2:178–182

[CR9] Bashtannik SV (2007) Зeмлeдeльчecкaя кyльтypa Южнoгo Кaзaxcтaнa эпoxи cpeднeвeкoвья (Agricultural culture of South Kazakhstan in the Middle Ages). КeмГУКИ (KemGUKI, Kemerovo State University of Culture and Arts), Kemerov

[CR10] Belenitsky AM, Bentovich IB, Bolshakov OG (1973) Cpeднeвeкoвый гopoд Cpeднeй Aзии (Medieval city of Central Asia). Nauka, Leningrad

[CR11] Billings TN, Cerasetti B, Forni L et al (2022) Agriculture in the Karakum: an archaeobotanical analysis from Togolok 1, southern Turkmenistan (ca. 2300–1700 B.C.). Front Ecol Evol 10:995490. 10.3389/fevo.2022.995490

[CR12] Bosworth CE, Asimov MS (2000) History of civilizations of Central Asia, Vol 4: The Age of achievement, A.D. 750 to the end of the fifteenth century; Part Two: The Achievements. UNESCO Publishing, Paris

[CR13] Bretschneider E (1888) The Travels of Ch’ang Ch’un to the West, 1220–1223 recorded by his disciple Li Chi Ch’ang. In: Bretschneider E (ed) Mediaeval Researches from Eastern Asiatic Sources. Trübner & Co, London, pp 35–199

[CR14] Brite EB (2016) Irrigation in the Khorezm oasis, past and present: a political ecology perspective. J Polit Ecol 23:1–25. 10.2458/v23i1.20177

[CR15] Brite EB, Khozhaniyazov G, Marston JM et al (2017) Kara-tepe, Karakalpakstan: Agropastoralism in a Central Eurasian Oasis in the 4th/5th century A.D. Transition J Field Archaeol 42:514–529. 10.1080/00934690.2017.1365563

[CR16] Browicz K (1982) Distribution of species from the genus *Rhus* L. in the eastern Mediterranean region and in southwestern Asia. Arboretum Kórnickie Rocznik 26:3–14

[CR17] Bubnova M (1987) Boпpocy o Зeмлeдeлии Ha Зaпaднoм Пaмиpe B IX-XI вв (Regarding the question of agriculture in the Western Pamirs in the IX-XI centuries). In: Ranov VA (ed) Пpoшлoe Cpeднeй Aзии: Apxeoлoгия, Hyмизмaтикa и Эпигpaикa, Этнoгpaния (The past of Central Asia: archaeology, numismatics and epigraphy, ethnography). Дoниш (Donish), Dushanbe, pp 59–66

[CR18] Buryakov YF (1990) Дpeвний и cpeднeвeкoвый гopoд Bocтoчнoгo Maвepaннaxpa. Шapyxия I-XVIII вв. н.э. (Ancient and medieval city of Eastern Maverannahr. Sharukhiya 1^st^–18th centuries AD). FAN, Tashkent

[CR19] Chang C (2018) Rethinking prehistoric Central Asia: Shepherds, Farmers, and Nomads. Routledge, New York

[CR20] Charbonnier J, Hopper K (2018) The *Qanāt*: a multidisciplinary and diachronic approach to the study of groundwater catchment systems in archaeology. Water Hist 10:3–11. 10.1007/s12685-018-0214-9

[CR21] Chen G, Zhou X, Wang J et al (2020) Kushan Period rice in the Amu Darya Basin: evidence for prehistoric exchange along the southern Himalaya. Sci China Earth Sci 63:841–851. 10.1007/s11430-019-9585-2

[CR22] Childe GV (1964) What happened in history. Penguin Books, Baltimore

[CR23] Clarke D, Sala R, Deom J-M, Meseth E (2005) Reconstructing irrigation at Otrar Oasis, Kazakhstan, AD 800–1700. Irrig Drain 54:375–388. 10.1002/ird.195

[CR24] Dal Martello R, von Baeyer M, Hudson M et al (2023) The domestication and dispersal of large-fruiting *Prunus* spp.: a metadata analysis of archaeobotanical material. Agronomy. 10.3390/agronomy13041027

[CR25] Danilevsky VV, Kokonov VN, Nikitin VA (1940) Иccлeдoвaниe pacтитeльныx ocтaткoв из pacкoпoк coгдийcкoгo зaмкa VIII вeкa нa гope Myг в Taджикиcтaнe (A Study of the plant remains excavated from the VIII century settlement of Mugh in Tajikistan). In: Komarov V (ed) Pacтитeльнocть Taджикиcтaнa и ee ocвoeниe (Vegetation of Tajikistan and Human Interaction). Tp. Taджикиcтaн бaзы AH CCCP (Tajikistan Base of Science of the SSSR), Dushanbe, pp 479–505

[CR26] De la Vaissière É (2017) Early medieval central Asian population estimates. J Econ Soc Hist Orient 60:788–817. 10.1163/15685209-12341438

[CR27] Decker M (2009) Plants and progress: rethinking the islamic agricultural revolution. J World Hist 20:187–206

[CR28] Del Cerro C, Córdoba JM (2018) Archaeology of a *falaj* in al Madam Plain (Sharjah, UAE); a study from the site. Water Hist 10:85–98. 10.1007/s12685-018-0210-0

[CR29] Dukhovny VA, de Schutter J (2011) Water in Central Asia: past, present, future. Taylor & Francis Group, London

[CR30] Fairbairn A, Asouti E, Near J, Martinoli D (2002) Macro-botanical evidence for plant use at Neolithic Çatalhöyük, south-central Anatolia, Turkey. Veget Hist Archaeobot 11:41–54

[CR31] Fairbairn AS, Wright NJ, Weeden M et al (2019) Ceremonial plant consumption at Middle Bronze Age Büklükale, Kırıkkale Province, central Turkey. Veget Hist Archaeobot 28:327–346. 10.1007/s00334-018-0703-x

[CR32] FAOSTAT (2022) Food and Agriculture Organization of the United Nations Database. https://www.fao.org/faostat/ru/#faq. Accessed 26 Feb 2022

[CR33] Frachetti MD (2012) Multiregional emergence of mobile pastoralism and nonuniform institutional complexity across Eurasia. Curr Anthropol 53:2–38. 10.1086/663692

[CR34] Frye R (2005) Ibn Fadlan’s Journey to Russia: a tenth-century traveler from Baghad to the Volga River. Markus Wiener Publishers, Princeton

[CR35] Fuks D, Amichay O, Weiss E (2020) Innovation or preservation? Abbasid aubergines, archaeobotany, and the Islamic Green Revolution. Archaeol Anthropol Sci 12:50. 10.1007/s12520-019-00959-5

[CR36] Fuller DQ, Stevens CJ (2019) Between domestication and civilization: the role of agriculture and arboriculture in the emergence of the first urban societies. Veget Hist Archaeobot 28:263–282. 10.1007/s00334-019-00727-4 10.1007/s00334-019-00727-4 PMC6499764 31118541

[CR37] Golden PB (2011) Central Asia in world history. Oxford University Press, Oxford

[CR38] Gorbunova NG (1986) The culture of ancient Ferghana: VI century B.C.-VI century A.D. BAR International Series 281. BAR Publishing, Oxford

[CR39] Grenet F (2002) Samarkand i. History and Archeology. Encyclopaedia Iranica online. 10.1163/2330-4804_EIRO_COM_185

[CR40] Haldane CW (1990) Shipwrecked plant remains. Biblic Archaeol 53:55–60. 10.2307/3210160

[CR41] Harris D (2010) Origins of agriculture in the Western Central Asia: an environmental-archaeological study. University of Pennsylvania Museum of Archaeology and Anthropology, Philadelphia

[CR42] Hawqal Ibn A al-QM (977) The oriental geography of Ebn Haukal: An Arabian traveller of the tenth century (AD 977). Translated from a manuscript in his own possession, collated with one preserved in the library of Eton College, by Sir William Ouseley in 1800. Oriental Press, London

[CR43] Hensellek B (2019) A Sogdian drinking game at Panjikent. Iran Stud 52:837–857. 10.1080/00210862.2019.1667224

[CR44] Herrmann G, Kurbansakhatov K (1994) The international merv project, preliminary report on the second season (1993). Iran 32:53–75

[CR45] Herrmann G, Masson VM, Kurbansakhatov K (1993) The international merv project, preliminary report on the first season (1992). Iran 31:39–62. 10.2307/4299886

[CR46] Jiang H, Wu Y, Wang H et al (2013) Ancient plant use at the site of Yuergou, Xinjiang, China: implications from desiccated and charred plant remains. Veget Hist Archaeobot 22:129–140. 10.1007/s00334-012-0365-z

[CR47] Johns J (1984) Review of “A green revolution? - agricultural innovation in the early islamic world the diffusion of crops and farming techniques, 700–1100 by Andrew M. Watson, Cambridge University Press 1983.” J Afr Hist 25:343–344. 10.1017/S0021853700028218

[CR48] Lee C-L, Coyle HM, Palmbach TM et al (2005) DNA Analysis of ingested tomato and pepper seeds. Am J Forensic Med Pathol 26:330–333. 10.1097/01.paf.0000188084.25905.3b 16304465 10.1097/01.paf.0000188084.25905.3b

[CR49] Lewis RA (1966) Early irrigation in West Turkestan. Ann Assoc Am Geogr 56:467–491. 10.1111/j.1467-8306.1966.tb00573.x

[CR50] Lisitsina GN (1969) The earliest irrigation in Turkmenia. Antiquity 43:279–288. 10.1017/S0003598X00040710

[CR51] Livšic VA (2009) Sogdian “Ancient Letters” (II, IV, V). Scrinium 5:344–352. 10.1163/18177565-90000228

[CR52] Malatesta LC, Castelltort S, Mantellini S et al (2012) Dating the irrigation system of the Samarkand oasis: a geoarchaeological study. Radiocarbon 54:91–105. 10.2458/azu_js_rc.v54i1.15839

[CR53] Mantellini S (2015) Irrigation systems in Samarkand. In: Selin H (ed) Encyclopaedia of the history of science, technology, and medicine in non-western cultures. Springer, Dordrecht, pp 1–14

[CR54] Mantellini S (2018) Landscape archaeology and irrigation systems in Central Asia: a view from Samarkand (Uzbekistan). In: Domenici D, Marchetti N (eds) Urbanized Landscapes in Early Syro-Mesopotamia and Prehispanic Mesoamerica: Papers of a Cross-Cultural Seminar held in Honour of Robert McCormick Adams. Harrassowitz, Wiesbaden, pp 169–204

[CR55] Mantellini S (2019) Urbanscape vs. Landscape, or Urbanscape as Landscape?: A case from ancient Samarkand (Sogdiana). In: Baumer C, Novák M (eds) Urban Cultures of Central Asia from the Bronze Age to the Karakhanids. Harrassowitz, Wiesbaden, pp 185–202

[CR56] Mantellini S, Rondelli B, Stride S (2008) Analytical Approach for Representing the Water Landscape Evolution in Samarkand Oasis (Uzbekistan). In: Jerem E, Redő F, Szeverényi V (eds) On the Road to Reconstructing the Past. Computer Applications and Quantitative Methods in Archaeology (CAA). Proceedings of the 36th International Conference. Budapest, April 2–6, 2008. Archaeolingua Foundation, Budapest, pp 387–396

[CR57] Mantellini S (2017) A city and its landscape across time: Samarkand in the ancient Sogdiana (Uzbekistan). In: Garagnani S, Gaucci A (eds) Knowledge, analysis and innovative methods for the study and the dissemination of ancient urban areas. Archeologia e Calcolatori 28.2. All’Insegna del Giglio, Sesto Fiorentino, pp 333–342

[CR58] Marshak BI, Raspopova VI (2016) Panjikent and the Arab Conquest. J Inner Asian Art Archaeol 7:255–275. 10.1484/J.JIAAA.4.2017013

[CR59] Miller N (1993) Preliminary archaeobotanical results from the 1989 excavation at the central Asian site of Gonur Depe, Turkmenistan. Information Bull 19:149–163

[CR60] Miller NF (1999) Agricultural development in western Central Asia in the Chalcolithic and Bronze Ages. Veget Hist Archaeobot 8:13–19

[CR61] Miller NF, Spengler RN, Frachetti M (2016) Millet cultivation across Eurasia: Origins, spread, and the influence of seasonal climate. Holocene 26:1,566-1,575. 10.1177/0959683616641742

[CR62] Miller NF (2011) Preliminary archaeobotanical results. In: Pollock S, Bernbeck R (eds) Excavations at Monjukli Depe, Meana-Čaača Region, Turkmenistan, 2010. Archäologische Mitteilungen aus Iran und Turan 43:169–237

[CR63] Mir-Makhamad B, Mirzaakhmedov S, Rahmonov H et al (2021) Qarakhanids on the Edge of the Bukhara Oasis: Archaeobotany of Medieval Paykend. Econ Bot 75:195–214. 10.1007/s12231-021-09531-6

[CR64] Mir-Makhamad B, Bjørn R, Stark S, Spengler RN (2022a) Pistachio (*Pistacia vera* L.) domestication and dispersal out of Central Asia. Agronomy 12:1758. 10.3390/agronomy12081758

[CR65] Mir-Makhamad B, Parshuto V, Lurje P et al (2022b) Зeмлeдeлиe в бacceйнe cpeднeгo Зepaвшaнa: пpeдвapитeльный oтчёт oб apxeoбoтaничecкиx иccлeдoвaнияx Пeнджикeнтa (Agriculture in the Middle Zarafshan Basin: A Preliminary Paleoethnobotanical Study from the Sogdian City of Panjakent). In: Lurje P, Semenov N (eds) Maтepиaлы Пeнджикeнтcкoй Apxeoлoгичecкoй Экcпeдиции. Bыпycк XXV (Materials of the Panjakent Archaeological Expedition. Issue 25). The State Hermitage Museum, Saint Petersburg, pp 65–74

[CR66] Mirzaachmedov D, Pozzi S, Adilov S et al (2019) Vardanzeh: The dynamics of settlement on the citadel based on the materials from medieval pottery complexes. In: Baumer C, Novák M (eds) Urban Cultures of Central Asia from the Bronze Age to the Karakhanids. Harrassowitz, Wiesbaden, pp 247–260

[CR67] Motuzaite Matuzeviciute G, Lightfoot E, O’Connell TC et al (2015) The extent of cereal cultivation among the Bronze Age to Turkic period societies of Kazakhstan determined using stable isotope analysis of bone collagen. J Archaeol Sci 59:23–34. 10.1016/j.jas.2015.03.029

[CR68] Motuzaite Matuzeviciute G, Preece RC, Wang S et al (2017) Ecology and subsistence at the Mesolithic and Bronze Age site of Aigyrzhal-2, Naryn valley, Kyrgyzstan. Quat Int 437:35–49. 10.1016/j.quaint.2015.06.065

[CR69] Motuzaite Matuzeviciute G, Abdykanova A, Kume S et al (2018) The effect of geographical margins on cereal grain size variation: Case study for highlands of Kyrgyzstan. J Archaeol Sci Rep 20:400–410. 10.1016/j.jasrep.2018.04.037

[CR70] Motuzaite Matuzeviciute G, Hermes TR, Mir-Makhamad B, Tabaldiev K (2020a) Southwest Asian cereal crops facilitated high-elevation agriculture in the central Tien Shan during the mid-third millennium BCE. PLoS ONE 15:e0229372. 10.1371/journal.pone.0229372 32433686 10.1371/journal.pone.0229372 PMC7239473

[CR71] Motuzaite Matuzeviciute G, Tabaldiev K, Hermes T et al (2020b) High-Altitude Agro-Pastoralism in the Kyrgyz Tien Shan: New Excavations of the Chap Farmstead (1065–825 cal b.c.). J Field Archaeol 45:29–45. 10.1080/00934690.2019.1672128

[CR72] Nakayama S, Akashi C (2019) アク・ベシム遺跡出土の植物遺存体 (Plant remains recovered from the Ak-Beshim site). Teikyo Univ Inst Cult Prop Res Rep 18:19–41

[CR73] Nakayama S, Akashi C (2020) アク・ベシム遺跡出土の植物遺存体分析 (Analysis of plant remains recovered from Ak-Beshim site, 2019 excavation season). Teikyo Univ Inst Cult Prop Res Rep 19:17–34

[CR74] Narshakhi ABM (943) Tarikh-i-Bukhara (The History of Bukhara), AD 943. Translated by Richard Frye in 1954. Crimson Printing Company, Cambridge, Massachusetts

[CR75] Negmatov NN, Pulatov UP, Khmenitsky SG (1973) Уpтaкypгaн и Tиpмизaктeпa (Urtakurgan and Tirmizaktepa). Дoниш (Donish), Dushanbe

[CR76] Omel’chenko AV (2019) New Excavations in the Paikend City-Site. The Sogdian Pottery Assemblage of the Hellenistic Period. In: Baumer C, Novák M (eds) Urban Cultures of Central Asia from the Bronze Age to the Karakhanids. Harrassowitz, Wiesbaden, pp 203–226. 10.2307/j.ctvrnfq57.17

[CR77] Popper K (1959) The logic of scientific discovery. Basic Books, New York

[CR78] Pulak C (2008) The Uluburun Shipwreck and Late Bronze Age Trade. In: Aruz J, Benzel K, Evans JM (eds) Beyond Babylon: Art, Trade, and Diplomacy in the Second Millennium B.C. Metropolitan Museum of Art, New York, NY, pp 288–305

[CR79] Rapen C (2010) Oт Кoктeпe дo Киндикли-тeпe: Hoвый пoдxoд к иcтpии вoзникнoвния иppигaции в ceвepнoй чacти Зepaвшaнcкoй дoлины (From Koktepe to Kendekli-tepe: A new approach to the history of irrigation in the northern Zerefsahn). In: Alimova D, Amanbaeva B, Baipakov K et al (eds) Цивилизaции и кyльтypы Цeнтpaльнoй Aзии в eдинcтвe и мнoгooбpaзии, Maтepиaлы Meждyнapoднoй кoнфepeнции Caмapкaнд, 7–8 ceнтябpя 2009 г. (Civilizations and cultures of Central Asia in unity and diversity. Proceedings of the International Conference, 7–8 September, 2009). Meждyнapoдный Инcтитyт Цeнтpaльнoaзиaтcкиx иccлeдoвaний (International Institute for Central Asian Studies), Samarkand, pp 139–150

[CR80] Sala R, Deom J-M (2010) Medieval tortkuls of northern Tienshan and mid-low Syrdarya. In: Erofeeva I, Zhanaev B, Masanova L (eds) The role of the Eurasian steppes nomads in the development of world military art, scientific readings in commemoration of N. F. Masanov. Proceedings of the international conference Almaty 22–23 April 2010. Almaty, pp 263–286

[CR81] Shishkina GV (1994) Ancient Samarkand: Capital of Soghd. Bulletin of the Asia Institut 8:81–99

[CR82] Shishkina GV (1979) Глaзypoвaннaя кepaмикa Coгдa (Glazed ceramics of Sogd). FAN, Tashkent

[CR83] Spengler RN (2015) Agriculture in the Central Asian Bronze Age. J World Prehist 28:215–253. 10.1007/s10963-015-9087-3

[CR84] Spengler RN (2019) Fruit from the Sands: the Silk Road Origins of the Food we eat. University of California Press, Oakland

[CR85] Spengler RN (2021) Niche construction theory in archaeology: a critical review. J Archaeol Method Theory 28:925–955. 10.1007/s10816-021-09528-4

[CR86] Spengler RN, Willcox G (2013) Archaeobotanical results from Sarazm, Tajikistan, an Early Bronze Age Settlement on the edge: Agriculture and exchange. Environ Archaeol 18:211–221. 10.1179/1749631413Y.0000000008

[CR87] Spengler RN, Frachetti MD, Doumani PN (2014) Late Bronze Age agriculture at Tasbas in the Dzhungar Mountains of eastern Kazakhstan. Quat Int 348:147–157. 10.1016/j.quaint.2014.03.039

[CR88] Spengler RN, Ryabogina N, Tarasov PE, Wagner M (2016) The spread of agriculture into northern Central Asia: Timing, pathways, and environmental feedbacks. Holocene 26:1,527-1,540. 10.1177/0959683616641739

[CR89] Spengler RN, Miller NF, Neef R et al (2017) Linking agriculture and exchange to social developments of the Central Asian Iron Age. J Anthropol Archaeol 48:295–308. 10.1016/j.jaa.2017.09.002

[CR90] Spengler RN, de Nigris I, Cerasetti B et al (2018a) The breadth of dietary economy in Bronze Age Central Asia: Case study from Adji Kui 1 in the Murghab region of Turkmenistan. J Archaeol Sci Rep 22:372–381. 10.1016/j.jasrep.2016.03.029

[CR91] Spengler RN, Maksudov F, Bullion E et al (2018b) Arboreal crops on the medieval Silk Road: Archaeobotanical studies at Tashbulak. PLoS ONE 13:e0201409. 10.1371/journal.pone.0201409 30106958 10.1371/journal.pone.0201409 PMC6091944

[CR92] Spengler RN, Miller AV, Schmaus T et al (2021a) An imagined past?: nomadic narratives in Central Asian Archaeology. Curr Anthropol 62:251–286. 10.1086/714245

[CR93] Spengler RN, Stark S, Zhou X et al (2021b) A journey to the west: the ancient dispersal of rice out of East Asia. Rice 14:83. 10.1186/s12284-021-00518-4 34564763 10.1186/s12284-021-00518-4 PMC8464642

[CR94] Spengler RN, Tang L, Nayak A et al (2021c) The southern Central Asian mountains as an ancient agricultural mixing zone: new archaeobotanical data from Barikot in the Swat valley of Pakistan. Veget Hist Archaeobot 30:463–476. 10.1007/s00334-020-00798-8

[CR95] Squatriti P (2014) Of seeds, seasons, and seas: Andrew Watson’s medieval agrarian revolution forty years later. J Econ Hist 74:1,205-1,220. 10.1017/S0022050714000904

[CR96] Stark S, Kidd FJ, Mirzaakhmedov JK et al (2022) The Uzbek-American Expedition in Bukhara. Preliminary Report on the Third Season (2017). Iran 60:149–199. 10.1080/05786967.2020.1769495

[CR97] Stride S, Rondelli B, Mantellini S (2009) Canals versus horses: political power in the oasis of Samarkand. World Archaeol 41:73–87. 10.1080/00438240802655302

[CR98] Taylor WTT, Pruvost M, Posth C et al (2021) Evidence for early dispersal of domestic sheep into Central Asia. Nat Hum Behav 5:1,169-1,179. 10.1038/s41562-021-01083-y 33833423 10.1038/s41562-021-01083-y

[CR99] Tolstov S (1948) Дpeвний Xopeзм (Ancient Khoresm). MGU, Moskow

[CR100] Toonen WHJ, Macklin MG, Dawkes G et al (2020) A hydromorphic reevaluation of the forgotten river civilizations of Central Asia. Proc Natl Acad Sci USA 117:32,982-32,988. 10.1073/pnas.2009553117 10.1073/pnas.2009553117 PMC7776828 33318206

[CR101] Usmanova Z (1963) Эpк-Кaлa (Erk-Kala). In: Masson M (ed) Tpyды Южнo-Typкмeниcтaнcкoй apxeoлoгичecкoй кoмплeкcнoй экcпeдиции ЮTAКЭ (Proceedings of the South-Turkmenistan Archaeological Complex Expedition STACE). Academy of Science of the Turkmen Soviet Socialist Republic, Ashkhabat, pp 20–94

[CR102] De la Vaissière É (2005) Sogdian traders: a history. Translated by James Ward. Brill, Leiden

[CR103] Van der Veen M (2011) Consumption, Trade and Innovation: Exploring the Botanical Remains from the Roman and Islamic Ports at Quseir al-Qadim. Africa Magna Verlag, Frankfurt am Main, Egypt

[CR104] Vasil’ev AI (1934) Coгдийcкий зaмoк нa гope Myг (The Castle on Mount Mugh). In: Krachkovskii I, Freiman A (eds) Coгдийcкий cбopник (Sogdian collection). The USSR Academy of Science, Leningrad, pp 18–32

[CR105] Vinokurova M (1957) Tкaни из зaмкa нa гope Myг (Textile from the Mugh castle). In: Bentovich I (ed) Извecтия OOH AH Taджикcкoй CCP №14. Academy of Science in TajikSSR, Dushanbe, pp 17–32

[CR106] Vyatkin V (1926) Aфpacиaб - гopoдищe былoгo Caмapкaндa. Apxeoлoгичecкий oчepк (Afrasiab - a settlement of the former Samarkand. Archaeological essay). GlavNauka, Tashkent

[CR107] Watson AM (1974) The Arab agricultural revolution and its diffusion, 700–1100. J Econ Hist 34:8–35

[CR108] Watson AM (1983) Agricultural innovations in the early Islamic world. Cambridge University Press, Cambridge

[CR109] Yakubov Y (1979) Пapгap B VII-VIII вв. н. э.: Bepxний Зepaвшaн в эпoxy paннeгo cpeднeвeкoвья (Pargar in the VII-VIII c. A.D.: The upper Zarafshan in the Early Middle Ages). Donish, Dushanbe

[CR110] Yang Q, Zhou X, Spengler RN et al (2020) Prehistoric agriculture and social structure in the southwestern Tarim Basin: multiproxy analyses at Wupaer. Sci Rep 10:14235. 10.1038/s41598-020-70515-y 32859982 10.1038/s41598-020-70515-y PMC7455698

[CR111] Zhou X, Yu J, Spengler RN et al (2020) 5,200-year-old cereal grains from the eastern Altai Mountains redate the trans-Eurasian crop exchange. Nat Plants 6:78–87. 10.1038/s41477-019-0581-y 32055044 10.1038/s41477-019-0581-y

[CR112] Zohary D, Hopf M, Weiss E (2012) Domestication of plants in the old world. Oxford University Press, Oxford

