# Supplementary material for: Testing the applicability of Watson’s Green Revolution concept in first millennium ce Central Asia
Source: Veg Hist Archaeobot. 2023 May 12;35(1):143–55. doi: 10.1007/s00334-023-00924-2 (PMC12881048; doi:10.1007/s00334-023-00924-2)
Supplement: Supplementary file 3 — Supplementary file3 (DOCX 30 KB) [file 334_2023_924_MOESM3_ESM.docx]

**Supplementary Material 3**

**Testing the applicability of Watson’s Green Revolution concept in first millennium CE Central Asia**

Basira Mir-Makhamad^1,2,3^* and Robert N. Spengler III^1,3^

1. Department of Archaeology, Max Planck Institute of Geoanthropology, Jena, Germany
2. Ancient Oriental Studies Department, Friedrich Schiller University, Jena, Germany
3. Domestication and Anthropogenic Evolution Research Group, Max Planck Institute of Geoanthropology, Jena, Germany

Basira Mir-Makhamad <https://orcid.org/0000-0002-1414-0392>

Robert N. Spengler III <https://orcid.org/0000-0002-5648-6930>

Corresponding author: mirmakhamad@shh.mpg.de

**Central Asia and the Islamic conquests**

The modern area of Central Asia – from the Caspian Sea to the western China – is bound by Russia to the north and by Iran and Afghanistan to the south (Fig. 1). Most of the geographic borders of this region are rather recent in origin and Central Asia has had a dynamic history and prehistory, with continual population turnovers and historically documented imperial expansions (Damgaard et al. 2018; Zhang et al. 2021). Central Asia is an extremely ecologically and culturally diverse region, and it is not possible to cover all of this diversity in this paper. Therefore, we focus on the central regions of Central Asia, Sogdiana (Transoxiana). Further to the south, along the Kopet Dag Mountains, the earliest urban centers date to the fourth millennium BCE but urbanization in the core areas of Central Asia appears to have started in the first millennium BCE (Belinskij et al. 1973); while there are still debates (e.g. Kidd and Stark 2019). While Watson’s model presumably works better in the southern regions, we choose to focus on what would have been the extreme peripheries of the early caliphate. Additionally, the study of the oases of pre-Islamic southern Central Asia, including Khorezm, Sogdiana, Margiana, and Bactria, is complicated by limited excavation of levels dating to the Antique-period (sixth century BCE – third century CE) (e.g. Kidd and Stark 2019).

Starting with the Arab military campaigns in Transoxiana (the region between the rivers of the Amu-Darya to the south and the Syr-Darya to the north) at the end of seventh and beginning of eighth centuries CE, Central Asia was gradually Islamized, shifting from a mix of Buddhism, Nestorian Christianity, and Zoroastrianism. The political conquests and conversion to Arabic were slow moving, starting from the large urban centers in the south and moving north over several decades, from Merv, Paykend, Bukhara, Samarkand, Chach, Khohanda, and later the Jetysuu area in CE 751 (Abazov 2008). Islam became the dominant religion across Transoxiana by the second half of the eighth century CE; however, it took several more centuries before much of the rest of Central Asia converted, with the Turkic-speaking Qarakhanid (CE 999 – 1220) elites supposedly converting soon after pushing the Samanids (CE 875 – 999) out of power (Golden 2011). Most historians accept that the period after the establishment of the new political authority was marked by greater centralized power and investment in the arts and sciences. Starr (2013) articulated, in *Lost Enlightenment*, that many prominent Islamic scholars, including Muhammad ibn Musa al-Khwarizmi, Abu Nasr Muhammad al-Farabi, and Ahmad al-Farghani, came from Central Asia during this period.

The Islamic cultural expansion does not appear to have been accompanied by any sharp and significant mark on the pool of paternal lineages, as demonstrated in recent genetic studies in Transoxiana (Zhabagin et al. 2017). The major population division within Central Asia remained a north/south divide with more pastoralism and (presumably) Turkic-speaking people on the northern steppe and in the coniferous forest belt and agricultural villages in the mountain foothills and desert oases. De la Vaissiere (2017, p. 815) evaluating historical sources and archaeological data, proposed that, “Western Central Asia was five to ten times heavily more populated than Eastern Central Asia” in the eighth century CE. Additionally, the level of centralization in political authority was different among differing ecological zones.

**Environmental Diversity**

Central Asia is characterized by an arid or semi-arid intercontinental climate, with cold winters and dry and hot summers, depending on the geographic zone. The region consists of a diverse array of microclimatic pockets, including: foothills; fertile river basins and oases; high mountain pastures; western dry deserts; steppes; and a taiga forest belt in the north. Temperatures vary significantly based on elevation; desert zones in western Uzbekistan and Turkmenistan (the Karakum and Kyzylkum deserts) have very cold winters and extremely hot summers. Mild climates are prominent in the fertile river basins and oases, where, historically, sedentary farming populations were prevalent. Foothill zones are the best ecological niche for arboriculture since the climate is also relatively mild there.

**References**

Abazov R (2008) Palgrave concise historical atlas of Central Asia. Macmillan, London

Belinskij A, Bentovich I, Bol’shakov O (1973) Средневековый город Средней Азии. Nauka, Leningrad

Damgaard P de B, Marchi N, Rasmussen S, et al (2018) 137 ancient human genomes from across the Eurasian steppes. Nature 557:369–374. https://doi.org/10.1038/s41586-018-0094-2

de la Vaissière É (2017) Early Medieval Central Asian Population Estimates. J Econ Soc Hist Orient 60:788–817. https://doi.org/10.1163/15685209-12341438

Golden P (2011) Central Asia in World History. Oxford University Press, Oxford/New York

Kidd F, Stark S (2019) Urbanism in Antique Sogdiana? In: Urban Cultures of Central Asia from the Bronze Age to the Karakhanids. Harrassowitz Verlag, Wiesbaden, pp 163–184

Starr SF (2013) Lost enlightenment: Central Asia’s golden age from the Arab conquest to Tamerlane. Princeton University Press, New Jersey

Zhabagin M, Balanovska E, Sabitov Z, et al (2017) The Connection of the Genetic, Cultural and Geographic Landscapes of Transoxiana. Sci Rep 7:3085.
https://doi.org/10.1038/s41598-017-03176-z

Zhang F, Ning C, Scott A, et al (2021) The genomic origins of the Bronze Age Tarim Basin mummies. Nature 599:256–261. https://doi.org/10.1038/s41586-021-04052-7
